# Myoinositol: The Bridge (PONTI) to Reach a Healthy Pregnancy

**DOI:** 10.1155/2017/5846286

**Published:** 2017-01-15

**Authors:** Pietro Cavalli, Elena Ronda

**Affiliations:** Clinical Genetics, ASST Cremona, Via Concordia 1, 26100 Cremona, Italy

## Abstract

The use of folic acid in the periconceptional period can prevent about 70% of neural tube defects (NTDs). In the remaining cases, no medical prevention is available, and those conditions should be defined as folate-resistant NTDs. Rodent models suggest that some folate-resistant NTDs can be prevented by inositol (myoinositol and chiroinositol) supplementation prior to pregnancy. Should folic acid be combined with myoinositol periconceptional supplementation to reduce the overall risk of NTDs even in humans? Hereafter, we discuss the results from the PONTI study that strongly support both the effectiveness and safety of myoinositol periconceptional supplementation in preventing human NTDs. We further report on the largest case series of pregnancies treated with myoinositol and folic acid. At our institution, a sequential study during 12 years involved mothers at risk of fetal NTDs, and 29 babies from 27 pregnancies were born after periconceptional combined myoinositol and folic acid supplementation. No case of NTDs was observed, despite the high recurrence risk in the mothers. Taken together, those data suggest that periconceptional folic acid plus myoinositol can reduce both the occurrence and recurrence risks of NTDs in a greater number of cases than folic acid alone.

## 1. Background

According to EUROCAT (European Surveillance of Congenital Anomalies) data, the EU prevalence of major congenital abnormalities is 23.9 per 1000 births. Among those defects, the frequency of nervous system defects has been calculated at 2.3 per 1000, and the prevalence of neural tube defects (NTDs) is 0.9 per 1000 (years 2003–2007) [1]. The neural tube is an embryonic structure that gives rise to the Central Nervous System (CNS) and is completely closed in the fourth week of gestation. An incomplete or no closure causes a severe anomaly of the CNS, and the subsequent exposure to the amniotic fluid environment leads to neurodegeneration and to severe damage of the exposed brain and/or spinal cord [2].

In most European countries, prenatal diagnosis of NTDs is easily available by ultrasound examination, and about 70% of spina bifida and 83% of anencephaly cases are prenatally identified, most of them leading to termination of pregnancy [3, 4].

So, it should be argued that NTDs seemingly became an “invisible” problem, as rarely seen in the newborn. However, the problem related to NTDs is far to be resolved and comes back in through the window, since the need of estimating the risk of recurrence for the next pregnancies is required for each affected pregnancy and reducing that risk should be the goal of every medical intervention.

Periconceptional folic acid supplementation has proved able to reduce the risk of NTDs up to 70% (occurrence risk: OR 0.28, CI 95% 0.15–0.53; recurrence risk: 0.68, CI 95% 0.17–0.60) [5].

Folic acid fortification also reduces the risk of NTDs (OR 0.54, CI 95% 0.46–0.63) [6]. Given those data, it comes without saying that about 30% of NTDs should be considered “resistant” to folic acid fortification and supplementation, and no intervention is available in those cases that are resistant to folic acid intake. The present paper deals with the problem of preventing that particular class of NTDs (folate-resistant NTDs) through the therapeutic use of inositol, a cyclic carbohydrate with a 6-carbon ring structure. Inositol is found widely throughout mammalian tissues as phosphatidylinositol and in cell membranes as phosphoinositide. It is no longer considered a member of the vitamin B complex, as it was in the past, since it can be produced by the human body from glucose. Inositol is a fundamental nutrient required by human cells in culture to grow and survive.

## 2. Inositol and Neural Tube Defects: Experimental Models

The relation between NTDs and inositol was first suggested in 1988 in rat embryos [7]. After that preliminary observation, the role of inositol in embryonic development and its effects on embryogenesis were further explored in the early 1990s, using rodent models. The supplementation with inositol of mouse embryo cultures exposed to an excess of glucose was shown to reduce the incidence of neural tube defects, normalizing the closure of the anterior neural tube. Indomethacin, an inhibitor of cyclooxygenase-1 and cyclooxygenase-2 which are involved in arachidonic acid metabolism and prostaglandin synthesis, can reverse the favorable effects of inositol on neural tube closure. Moreover, the addition of prostaglandin E2 to those embryo cultures leads to the normal development and closure of the neural tube, suggesting a protective role of both inositol and arachidonic acid pathway against CNS defects associated with diabetic embryopathy [8]. At the same time, an inverse correlation between glucose concentration and inositol content in rat embryo cultures was demonstrated, as well as a significant reduction of neural defects after inositol supplementation [9]. Following those seminal observations, the relationship between inositol and NTDs was confirmed in curly tail mice a few years later [10]. The curly tail mutant mouse has been used since 1976 as an experimental model to study NTDs [11], as the homozygous mutant ct (ct−/ct−) mouse is associated with NTD phenotypes (spina bifida, curly tail, and exencephaly), although characterized by variable expression and incomplete penetrance.

Of note, inositol deficiency is associated with an increased frequency of NTDs in the curly tail embryos in vitro [12].

Spina bifida in curly tail mice (ct−/ct−) results from a defective closure of the posterior neuropore, which forms the caudal region of the neural tube. The balance between the anterior and posterior growths of embryonic tissues reflects on the angle of curvature of the body axis.

In (ct−/ct−) mice, both the posterior endoderm and the notochord grow slowly, and the unbalanced growth between ventral and dorsal tissue causes an excessive curvature of the caudal region, which in turn induces a delay in the closure of the posterior neuropore, resulting in the curly tail phenotype, or in more severe closure defect, represented by spina bifida. A balanced embryonic tissue growth can normalize the curvature of the body axis, thus reducing the frequency of spina bifida. In the curly tail model, the neural tube closure can be normalized by the administration of inositol, which stimulates the growth of mesodermal tissues, thus restoring the normal growth of the embryo [13, 14].

Further evidence of the preventive effect of inositol on NTDs incidence is not restricted to curly tail mice, since protection against diabetes-induced NTDs has been observed in other rodent models.

Maternal diabetes is a condition associated with an increased risk of fetal malformations, and among them, NTDs are often observed.

Dietary inositol supplementation of 0.08 mg/kg/day is also effective in significantly decreasing the frequency of embryonic NTDs in diabetic rats [15, 16]. Of note, no evidence of an adverse effect of inositol either on pregnancy success or on fetal outcome was found in inositol-treated mice.

A more comprehensive approach revealed that the incidence of NTDs in the curly tail mutant mouse is not reduced by folic acid, suggesting that different subtypes of NTDs exist, some of them being resistant to folate intake.

As stated above, no more than 70% of human NTDs can be prevented by folic acid administration, so the curly tail mutant mouse was viewed as an experimental model to test the possibility of a combined treatment of folic acid plus inositol even in humans.

More recently, other findings strongly suggest that inositol metabolism is needed for normal brain and CNS development and that NTDs are associated with disruption of inositol signaling [17, 18].

Therefore, data from rodent models strongly support a distinct inositol-dependent metabolic pathway that, when stimulated, can prevent the cellular dysfunction leading to spinal NTDs.

## 3. Inositol and Neural Tube Defects: Humans

As inositol supplementation significantly reduces the incidence of spina bifida in the curly tail mouse, as well as in diabetic rats, the question is whether the risk of NTDs could be reduced also in humans and whether the risk of folate-resistant NTDs can be reduced by inositol treatment [19].

In humans, significantly lower inositol concentrations have been reported in the blood of mothers carrying NTD fetuses compared with normal pregnancies, and mothers with low blood levels of inositol show a 2.6-fold increased risk of an affected offspring [20].

Moreover, the association between maternal diabetes and the risk of fetal NTDs can be explained by the competitive effect of hyperglycemia on the transmembrane transportation of both arachidonic acid and inositol, suggesting that inositol supplementation could restore normal levels of cellular myoinositol and counteract the inhibition of neural tube closure caused by elevated glucose blood levels [8].

To answer the question whether inositol could be useful in reducing the recurrence risk of NTDs in human pregnancies, Cavalli and Copp proposed the first periconceptional treatment with myoinositol and folic acid to a woman with two previous NTD-affected pregnancies despite a correct folic acid intake. The recurrence of those congenital malformations was attributed to the resistance to folic acid, likely mimicking the curly tail experimental model.

That woman underwent supplementation with 500 mg myoinositol and 5 mg folic acid in the periconceptional period, and a healthy baby was born. Of note, no adverse effects on the mother and the fetus were associated with that treatment [21].

However, the effectiveness of inositol intake in reducing NTD risk has been questioned, as dietary inositol intake and NTD risk were not correlated statistically in a retrospective questionnaire analysis [22]. Still, the results from a questionnaire analysis cannot be considered as reliable as the measurement of inositol in blood or urine and should be taken cautiously. Moreover, it could be argued that inositol supplementation, but perhaps not normal dietary intake, could be beneficial in preventing folate-resistant NTDs in humans, as in rodents.

In any case, after the first pioneering result, a prospective pilot study was started aiming to demonstrate that women with at least one previous NTD-affected pregnancy despite correct folate intake and exposed to myoinositol in their periconceptional period (a) will not experience any adverse effects after inositol supplementation (1000 mg/day) and (b) will give birth to babies not affected by NTDs, so demonstrating a positive effect in reducing the recurrence risk.

Even in the absence of statistical significance, and given the difficulty to design and perform a randomized clinical trial (RCT), the prospective study demonstrated that myoinositol taken in the periconceptional period was not associated with collateral or side effects.

The study was performed on 15 pregnancies at risk of putative folate-resistant NTDs treated with inositol and folic acid (1000 mg myoinositol and 5 mg folic acid) in their periconceptional period, starting at least 60 days before conception and continued until the 8th week of pregnancy. All the women underwent correct folate supplementation in their previous affected pregnancies. Despite their high recurrence risk, all the babies were born without NTDs, and no adverse event was reported in that series [23].

## 4. Recent Results

Based on the results reported before, the PONTI (Prevention of Neural Tube Defects by Inositol) study was designed as a pilot study, aiming to demonstrate the feasibility of a randomized clinical trial (RCT) to evaluate the effectiveness of inositol plus folic acid in preventing a greater number of NTDs as compared to folic acid alone, according to the previous experimental hypothesis. The results from the PONTI study have been recently published [24].

The PONTI study confirmed the difficulty of enrolling the greater number of cases needed to perform an RCT, as well as the burden of obtaining significant results.

However, those results confirm a trend towards the association between inositol and folic acid intake and a reduced risk of NTDs.

Moreover, the PONTI study confirms the feasibility of a full-scale clinical trial on the use of inositol and folic acid to prevent a greater number of NTDs than folic acid alone.

Among the 117 women with a previous NTD-affected pregnancy and planning a new pregnancy that were contacted, 18 were ineligible, 52 declined randomization, 30 were lost at follow-up, and 33 were randomized for each arm (folic acid plus inositol versus folic acid plus placebo).

A corrigendum was added after publication, making the study results somewhat difficult to discuss. However, the final results are favorable and are described as follows:

- 14 women in the inositol arm gave birth to normal babies.
- One affected pregnancy was found in the control group (19 originally randomized).

24 pregnancies were reported among the 52 women who declined randomization, and 2 NTDs were born from women not taking inositol. Again, no NTD case was reported in women who underwent inositol supplementation.

Those results confirm the association between inositol and folic acid intake and a reduced risk of NTDs.

Of note, no adverse events were found in all pregnancies treated with inositol, nor in the control group, suggesting that periconceptional inositol supplementation should be considered safe for both the mothers and the fetuses. In our opinion, this is one of the most important results of the PONTI study, and the safety of inositol intake prior to pregnancy should be underlined.

Further interesting results came from the evaluation of inositol measurement by mass spectrometry assay, suggesting that measuring blood inositol levels is more accurate than measuring urinary inositol, still allowing monitoring the inositol supplementation.

Moreover, the favorable results obtained by the PONTI study highlight the need of a more extensive evaluation on the role of myoinositol in preventing a greater number of NTDs, as compared to folic acid alone, or, alternatively, to study the effects of myoinositol on some subtypes of NTDs (i.e., folate-resistant NTDs).

However, even if a randomized clinical trial (RCT) would be necessary to demonstrate a statistically significant reduction in the rate of NTDs after myoinositol supplementation, it should be noted that, similar to rare diseases, the scarcity of at-risk subjects represents a barrier to overcome for the design of RCTs, which could only be performed with a greater/international recruitment effort. To overcome those difficulties, the authors suggest the possibility of a different approach (sequential study design), in which women at risk of NTDs undergo inositol supplementation after a previous affected pregnancy, in which folic acid alone was not effective. Prospectively, this approach could be seen as unethical and very long to be carried on. Alternatively, we propose that all the women with a previous NTD-affected pregnancy despite correct folic acid supplementation might take FA plus MI in the next pregnancy. As stated before, nonresponsiveness to FA supplementation should define a particular NTD subtype, namely, folate-resistant NTDs.

At our institution, over the course of 12 years, 27 pregnancies at high risk of folate-resistant NTDs were treated with 5 mg folic acid plus 1000 mg myoinositol supplementation in the periconceptional period, starting at least four weeks before pregnancy. All the mothers had undergone correct folic acid supplementation in their previous affected pregnancy/pregnancies. Despite the high recurrence risk, after combined folic acid and myoinositol supplementation, 29 babies not affected by NTDs were born. According to the different recurrence risks after one or more affected pregnancies, the expected frequency of NTDs in that selected population was 2–8 affected cases.

As no NTDs case was observed in this series, these results strengthen the PONTI results. Moreover, our data are consistent even with an Evidence-Based Medicine (EBM) approach. In fact, between the observed results and myoinositol plus folic acid periconceptional supplementation, a close temporal relation and a strong relationship can be identified. Furthermore, given the biological mechanisms of inositol in neurogenesis, those results are clearly plausible, consistent, coherent, and specific [25]. Even if it seems difficult to combine the conclusions from a sequential study with those from a randomized clinical trial, both results strongly support a favorable effect of myoinositol supplementation in reducing the overall frequency of NTDs.

## 5. Conclusion

So, in light of the results reported before on the use of myoinositol in reducing the risk of recurrence of NTDs, we suggest that mothers with a previous NTD-affected pregnancy despite correct periconceptional folic acid intake should be considered at risk of folate-resistant NTDs and should be treated with myoinositol and folic acid in the next pregnancy.

In our experience, this approach has proved to be effective and without side effects on both the mothers and the fetuses for at least fifteen years.

In conclusion, after many years, attempts, and uncertainties, there are now many and consistent data that confirm the importance of myoinositol supplementation in the prevention of human NTDs, as well as the safety of that prophylactic approach. Moreover, given the lack of side effects associated with myoinositol periconceptional supplementation, we suggest that the administration of both folic acid and myoinositol should be further investigated even in normal pregnancies.
