# Corrigendum to “Myoinositol: The Bridge (PONTI) to Reach a Healthy Pregnancy”

**DOI:** 10.1155/2018/6050369

**Published:** 2018-09-12

**Authors:** Pietro Cavalli, Elena Ronda

**Affiliations:** Clinical Genetics, ASST Cremona, Via Concordia 1, 26100 Cremona, Italy

In the article titled “Myoinositol: The Bridge (PONTI) to Reach a Healthy Pregnancy” [1], the “Competing Interest” section should read as follows:
